# Characterization of 3'-untranslated region of the mouse GDNF gene

**DOI:** 10.1186/1471-2199-13-2

**Published:** 2012-01-17

**Authors:** Kentaro Oh-hashi, Yoko Hirata, Kazutoshi Kiuchi

**Affiliations:** 1 Department of Biomolecular Science, Faculty of Engineering, Gifu University, 1-1 Yanagido, Gifu 501-1193, Japan; 2 United Graduate School of Drug Discovery and Medical Information Sciences, Gifu University, 1-1 Yanagido, Gifu 501-1193, Japan

## Abstract

**Background:**

Glial cell line-derived neurotrophic factor (GDNF) is a potent survival factor for many cell types, and its expression is widespread both within and outside of the nervous system. The regulation of GDNF expression has been extensively investigated but is not fully understood.

**Results:**

Using a luciferase reporter assay, we identified the role of the 3'-untranslated region (3'-UTR) of the mouse GDNF gene in the regulation of gene expression. We focused on a well-conserved A- and T-rich region (approximately 200 bp in length), which is located approximately 1000 bp downstream of the stop codon in exon 4 of the gene and contains three typical AU-rich elements (AREs), AUUUA. Interestingly, these AREs are well conserved in several GDNF genes. By testing reporter constructs containing various regions and lengths of the 3'-UTR fused to the end of the luciferase gene, we demonstrated that the ARE-induced decrease in luciferase activity correlates with the attenuation of the mRNA stability. Furthermore, we found that several regions around the AREs in the 3'-UTR suppressed the luciferase activity. Moreover, the expression level of the GDNF protein was negligible in C6 glioma cells transfected with the ARE-containing GDNF expression vector.

**Conclusions:**

Our study is the first characterization of the possible role of AREs and other suppressive regions in the 3'-UTR in regulating the amounts of GDNF mRNA in C6 cells.

## Background

Glial cell line-derived neurotrophic factor (GDNF) was originally purified from rat B-49-conditioned medium and was characterized as a potent neurotrophic factor for culturing dopaminergic neurons from the developing substantia nigra [1]. GDNF is a distantly related member of the transforming growth factor-β (TGF-β) superfamily [2], and additional GDNF homologs have also been cloned [3-5]. GDNF expression is widespread in both the central and peripheral nervous systems, in addition to outside of the nervous system [6-9]. The targeted disruption of the mouse GDNF gene showed that GDNF plays a critical role in the development of both kidney and enteric neurons during embryogenesis [10,11]. GDNF possesses multifunctional properties that regulate the development and differentiation of a variety of cell lineages and acts as a neurotrophic factor for specific types of neurons in the nervous system. Accordingly, many investigators have reported the regulation of GDNF mRNA in various types of cells, such as astrocytes, microglial cells and macrophages, both *in vitro *and *in vivo *during tissue development and in pathophysiological states, including in response to inflammatory stimuli, ischemic/hypoxic insults and spinal cord injury [12-16]. However, the precise mechanisms regulating GDNF mRNA expression are not yet fully understood.

For many genes, the promoter and enhancer activities of their 5'-flanking regions and introns have been extensively characterized in the evaluation of gene expression regulation. However, the regulation of mRNA stability has also been demonstrated to play an important role in controlling gene expression [17]. In particular, a sequence rich in adenosine (A) and uridine (U), containing the AU-rich element (ARE), AUUUA, has been identified to regulate expression levels of mRNA. The ARE motif was first identified within the 3'-untranslated regions (3'-UTRs) of mRNAs encoding cytokines [18], and many genes have been predicted to produce ARE-containing mRNAs [19,20].

In this study, we focused on the regulatory role of AREs in the 3'-UTR of exon 4 in the mouse GDNF gene. In addition, the ARE-containing region of GDNF exon 4 fused to the end of the luciferase or the mouse GDNF coding region markedly diminished each expression.

## Results

**Figure 1:**
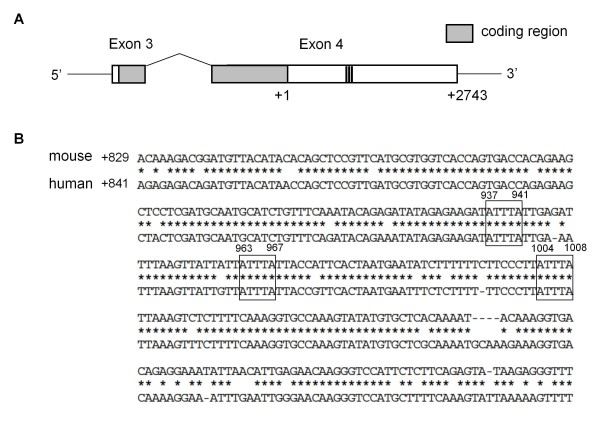
**Genomic structure of mouse GDNF**. A) Partial schematic diagram of the mouse GDNF gene. The coding regions are indicated by hatched boxes. In this study, the nucletotide immediatly after the stop codon in exon 4 is defined as +1. B) The nucleotide sequences of the A- and T-rich region of the mouse and human GDNF 3'-UTR. Nucleotide sequences in the A- and T-rich region conserved between the mouse and human GDNF 3'-UTR are indicated with asterisks.

To evaluate the characteristic features of the 3'-UTR of the mouse GDNF gene, which consists of an approximately 2800-bp long nucleotide sequence immediately following the stop codon (Figure 1A), we first compared the GDNF 3'-UTR among the following 11 species: *Danio rerio*, *Gallus gallus*, *Mus musculus*, *Rattus norvegicus*, *Canis lupus familiaris*, *Equus caballus*, *Bos taurus*, *Sus scrofa*, *Macaca mulatta*, *Pan troglodytes *and *Homo sapiens*. Except for the nucleotide sequences of the putative *D. rerio and G. gallus *GDNF 3'-UTRs, the sequences in the proximal half (approximately 1200 bp) and the end region (approxiately 200 bp) of the 3'-UTR are homologous to each other. Interestingly, an A/T-rich sequence, ATTTA [20], is well conserved among 11 species, and is serially located approximately 900 bp downstream of the stop codon in the GDNF 3'-UTR with the exception of *D. rerio*. As shown in Figure 1B, the homology of the nucleotide sequences of these A/T rich regions between mouse and human is approximately 90%. Therefore, we characterized the features of the well-conserved region of the mouse GDNF 3'-UTR, particularly the ARE consensus sequences, in regulating gene expression using a luciferase reporter system.

**Figure 2:**
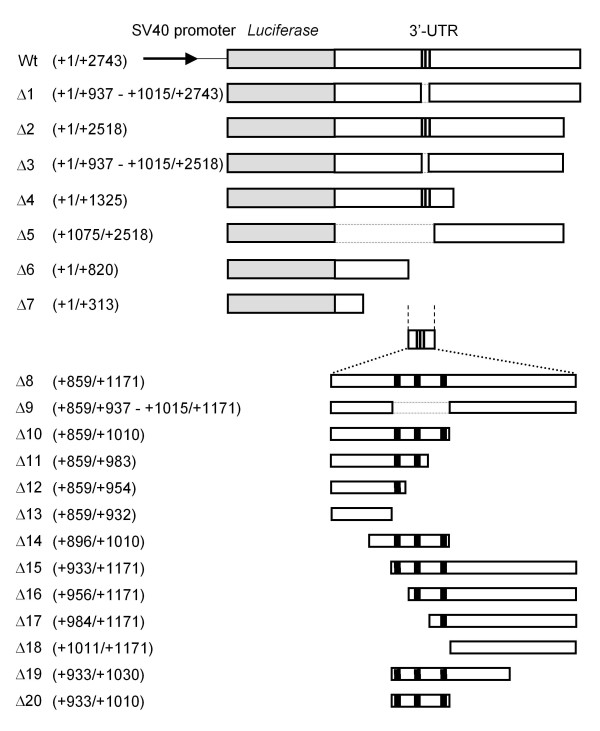
**Schematic structure of the ARE-containing reporter constructs used in this study**. Luciferase reporter constructs containing various regions and lengths of the mouse GDNF 3'-UTR at the end of the luciferase coding region. The bold line represents a typical ARE consensus sequence, AUUUA.

**Figure 3:**
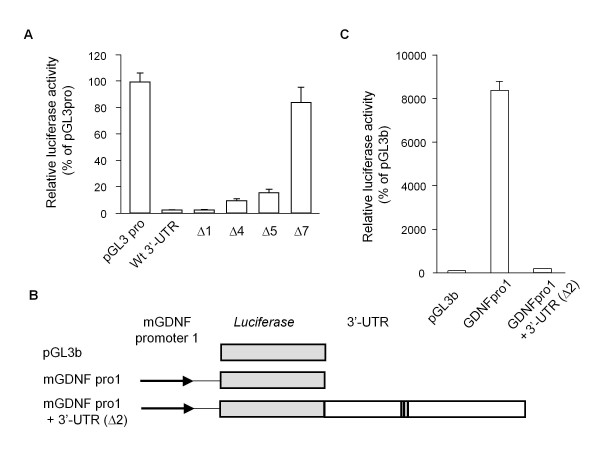
**The mouse GDNF 3'-UTR down-regulated luciferase activity in C6 cells**. Thirty-six hours after transfection with the pGL3-Promoter vector (pGL3pro) containing the indicated mouse GDNF 3'-UTR (A) or each mouse GDNF (mGDNF) promoter construct (B, C) containing the indicated length of the 3'-UTR, the luciferase activity was measured as described in the Materials and Methods. Values represent the means ± SD from 3 independent cultures, and the luciferase activity is expressed relative to that of the pGL3pro vector in panel A and to the pGL3-Basic vector (pGL3b) in panel C.

**Figure 4:**
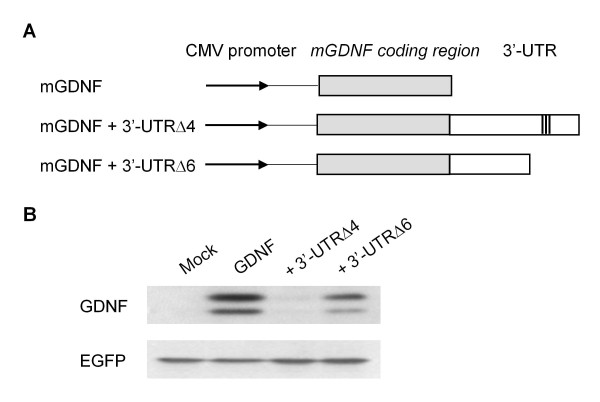
**The mouse GDNF 3'-UTR attenuated the amount of GDNF protein in C6 cells**. A) Schematic structure of the mouse GDNF coding construct containing the indicated length of the 3'-UTR. B) Thirty-six hours after co-transfection with each mouse GDNF construct and the pEGFP-N1 vector, the expression level of each protein was detected using Western blot analyses as described in the Materials and Methods.

As shown in Figure 2, we inserted the full-length mouse GDNF 3'-UTR into downstream of the luciferase gene in the pGL3-Promoter vector and estimated the luciferase activity in transiently transfected C6 glioma cells. The luciferase activity of the reporter gene containing the full-length mouse GDNF 3'-UTR was markedly lower than that of the control pGL3-Promoter vector without the 3'-UTR (Figure 3A). A series of deletion constructs of the 3' end of the mouse GDNF 3'-UTR (Δ1, Δ4, Δ5 and Δ7) revealed that the region around the ARE sequences showed suppressive effects on the expression of the reporter gene. In addition, the unconserved region in the distal half of the mouse GDNF 3'-UTR (Δ5) also suppressed luciferase activity. We also observed a similar inhibitory effect of the 3'-UTR (Δ2) on luciferase activity when the reporter constructs containing approximately 3.0 kb of the mouse GDNF promoter region were included in the pGL3 Basic vector (Figure 3B and 3C). Furthermore, the mouse GDNF construct that included the ARE sequences following the coding region led to marginal expression of the GDNF protein in transfected C6 glioma cells (Figure 4).

**Figure 5:**
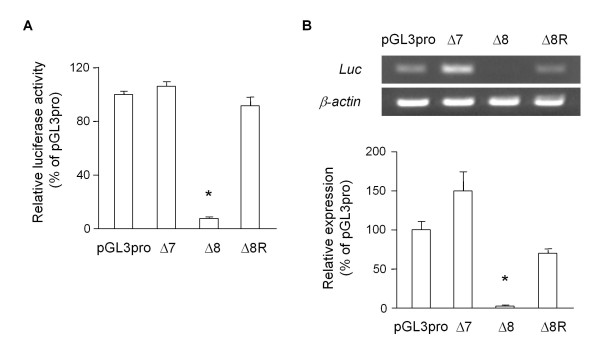
**ARE in the mouse GDNF 3'-UTR down-regulated luciferase mRNA in C6 cells**. A) Thirty-six hours after transfection with each GDNF promoter construct containing the indicated length of the 3'-UTR, the luciferase activity was measured as described in the Materials and Methods. Values represent the means ± SD from 3 independent cultures and are expressed relative to the luciferase activity of the pGL3-Promoter vector (pGL3pro). Δ8R represents a reporter construct in which the Δ8 fragment of the mouse GDNF 3'-UTR was inserted in the opposite direction. B) Thirty-six hours after transfection with the indicated reporter constructs, total RNA from each sample was extracted and subjected to RT-PCR as described in the Materials and Methods. The relative mRNA level of luciferase was calculated by the comparison to β-actin-normalized values of the mRNA level in pGL3-Promoter vector-transfected cells. Values represent the means ± SD from 3 independent cultures and are expressed relative to the luciferase activity (A) and mRNA (B) of the pGL3-Promoter vector. *p < 0.01, compared to the luciferase activity in the pGL3-Promoter vector-transfected cells (pGL3pro).

**Figure 6:**
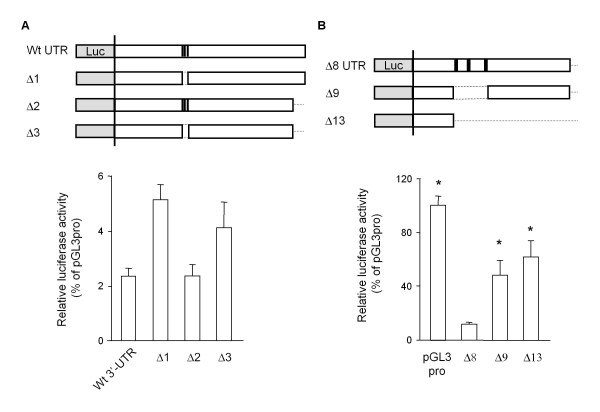
**Effect of the deletion of the core ARE on the luciferase activity in C6 cells**. Thirty-six hours after transfection with the indicated reporter constructs (A, B), the luciferase activity was measured as described in the Materials and Methods. Values represent the means ± SD from more than 3 independent cultures and are expressed relative to the luciferase activity of the pGL3-Promoter vector. *p < 0.01, compared to the luciferase activity in the Δ8 construct-transfected cells (Δ8).

**Figure 7:**
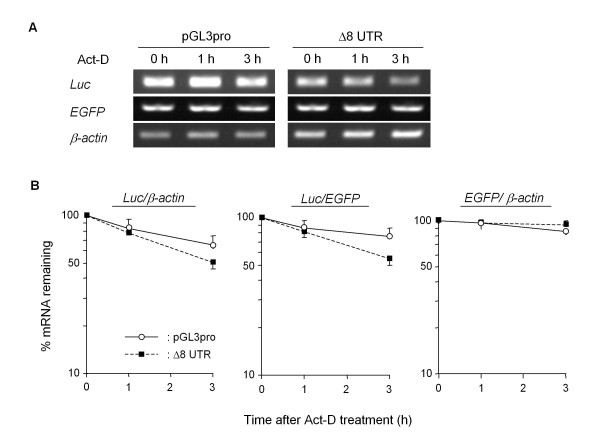
**ARE in the mouse GDNF 3'-UTR destabilized luciferase mRNA in C6 cells**. Thirty-six hours after transfection with the indicated reporter construct, together with the pEGFP-N1 vector, the cells were harvested (0 h, untreated cells) or treated with Act-D (5 μg/ml) for 1 or 3 h. The total RNA isolated from each sample was subjected to RT-PCR, and the relative mRNA levels of luciferase and EGFP were calculated by a comparison of β-actin or EGFP-normalized values with the level in the untreated cells (0 h) as described in the Materials and Methods (A, B). Values represent the means ± SD from four independent cultures.

By studying the suppressive feature in the well-conserved proximal half of the mouse GDNF 3'-UTR, we found that the insertion of an approximately 300 bp region of the 3'-UTR (+859/+1171) containing three AREs (Δ8) into the pGL3-Promoter vector was sufficient to suppress the luciferase activity in the C6 cells (Figure 5A). In contrast, the insertion of this suppressive region in the opposite direction (Δ8R) and the 3'-UTR (+1/+313) region of the same length (Δ7) into the pGL3-Promoter vector had little effect on the luciferase activity in the C6 cells. The expression of luciferase mRNA in the C6 cells transfected with the Δ8 construct was negligible compared to the cells transfected with the Δ7 or Δ8R construct (Figure 5B), and deletion of the core ARE region (+938/+1014) from the Wt, Δ2 and Δ8 constructs partialy recovered the promoter activity (Figure 6). The luciferase activity of the Wt or Δ2 construct in transfected cells was extremely low (approximately 2% of the pGL3pro-transfected cells), however the deletion of the core ARE region from the Wt and Δ2 constructs caused an approximately 2-fold increase in the promoter activity. The luciferase activity in the Δ9 construct-transfected cells was approximately half of that in the pGL3pro-transfected cells and was almost equal to that in the cells transfected with the Δ13 construct, which contains the upstream sequence (+859/+932) from the core ARE region only. Furthermore, we evaluated the stability of the luciferase mRNA in each case using Actinomycin D (Act-D) (Figure 7). To estimate the remaining luciferase mRNA in each of the reporter-transfected cell lines, we first determined the cycle count of the polymerase chain reaction (PCR) to detect the luciferase mRNA in the Δ8 construct-transfected cells. Three hours after treatment with Act-D, the amount of luciferase mRNA in the C6 cells transfected with the Δ8 construct was approximately 25% lower than that in the cells transfected with the original pGL3-Promoter vector; however, this difference was not significant.

**Figure 8:**
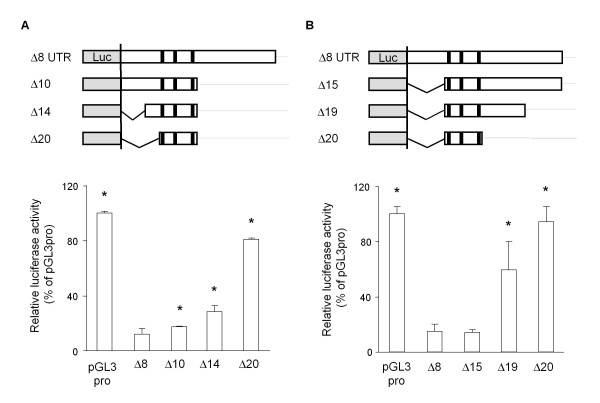
**Role of AU rich regions adjacent to the three AREs in down-regulating luciferase activity in C6 cells**. Thirty-six hours after transfection with the indicated reporter constructs (A, B), the luciferase activity was measured as described in the Materials and Methods. Values represent the means ± SD from more than 3 independent cultures and are expressed relative to the luciferase activity of the pGL3-Promoter vector. *p < 0.01, compared to the luciferase activity in the Δ8 construct-transfected cells (Δ8).

**Figure 9:**
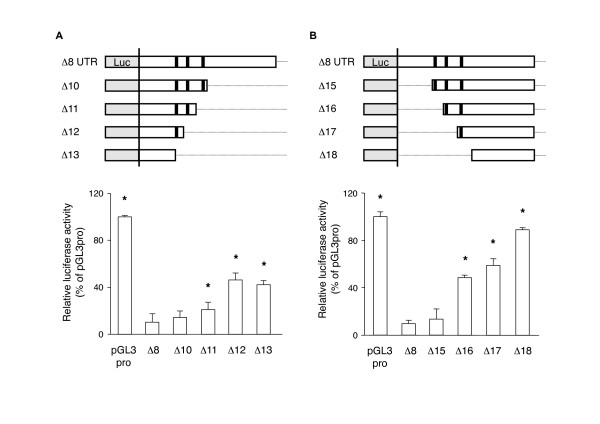
**Effect of three ARE on luciferase activity in C6 cells**. Thirty-six hours after transfection with the indicated reporter constructs (A, B), the luciferase activity was measured as described in the Materials and Methods. Values represent the means ± SD from 3 independent cultures and are expressed relative to the luciferase activity of the pGL3-Promoter vector. *p < 0.01, compared to the luciferase activity in the Δ8 construct-transfected cells (Δ8).

Lastly, we characterized the AREs of the mouse GDNF 3'-UTR and the nucleotide sequences adjacent to these elements by generating various deletion reporter constructs. The C6 cells transfected with the 3'-UTR construct containing the sequences from either side of the AREs (Δ10 and Δ15) showed lower luciferase activities; however, the cells transfected with the 3'-UTR construct without these adjacent sequences (Δ20) showed higher luciferase activity than both the Δ10 and Δ15 constructs (Figure 8). The C6 cells transfected with a construct of the 3'-UTR serially deleted from either side of the Δ8 region showed higher luciferase activities when the length of the Δ8 region was reduced and each ARE was sequentially deleted (Figure 9). Consistent with the results in Figure 6B, the Δ13 construct, which lacked the core ARE and following downstream regions (+933/+1171), still suppressed the lucifease activity by approximately 50%.

## Discussion

Using a luciferase reporter assay, our results are the first to demonstrate that the mouse GDNF 3'-UTR has multiple suppressive regions regulate gene expression. In this study, we employed three types of promoters (SV40, CMV and the intrinsic mouse GDNF promoter) and two genes (luciferase and GDNF) to characterize the features of the mouse GDNF 3'-UTR. Among several regions in the mouse GDNF 3'-UTR, we focused on the role of the AREs in the middle region of the 3'-UTR in regulating gene expression in a post-transcriptional manner, such as through mRNA stability, because these particular AREs are higly conserved among eleven different organisms. In our experiments, the C6 cells transfected with an expression construct in which the AU-rich region was immediately downstream of the coding region expressed negligible amounts of mRNA and protein. These results suggest that the suppressive effects of this AU-rich region in the mouse GDNF 3'-UTR are not affected by the coding sequences or promoters. Barreau *et al. *have proposed that there are three different classes of AREs [20], and according to their classification, the AU-rich region in the GDNF 3'-UTR belongs to Class I because it contains the ARE consensus sequences, AUUUA. In our deletion analyses, the reporters in which the AREs were serially deleted gradually lost their suppressive properties, demonstrating that three ARE consensus sequences in the mouse GDNF 3'-UTR play a cooperative role in regulating the amounts of mRNA. However, our results also indicated that this suppressive function does not simply depend on these conserved AREs. The Δ20 construct containing only the core ARE region (+933/+1010) did not exhibit a convincing suppressive effect. The Δ13 construct containing only the short region (+859/+932), just upstream from the ARE region, still exhibited reduced luciferase activity of approximately 50% compared to pGL3pro. It seems that both region adjacent to the AREs might be required to suppress the luciferase activity effectively. Hajarnis *et al. *have reported that the GC-rich sequences adjacent to an ARE in the 3'-UTR of phosphoenolpyruvate carboxykinase also function to destabilize the mRNA [21]. We also demonstrated that the core ARE-deletion (+938/+1014) from entire GDNF 3'-UTR caused an apparent doubling of luciferase activity. It is possible that other suppressive factors recognize currently uncharacterized regions in the mosue GDNF 3'-UTR to exert the full suppressive effect. In contrast to the high conservation of the region around the AREs among 11 species, the distal half of the mouse GDNF 3'-UTR (+1075/+2518) is approximately 85% homologous to only the putative rat GDNF 3'-UTR. Meanwhile, the posterior half of the human GDNF 3'-UTR is highly similar to only the putative Rhesus and chimpanzee sequences. The homology is lower between rodents and primates. As shown in Figures 2, 3 and 6, this unconserved region showed a marked suppressive effect on the promoter activity, and the deletion of the core ARE from the Wt and Δ2 constructs recovered the activity to a lesser extent. Therefore, we conclude that in addition to our characteirzed AREs, common and species-specific negative factors cooperatively recognize the concensus sequences in the GDNF 3'-UTR to regulate the expression of the GDNF gene. However, the precise mechanism for the down-regulation of expression by the 3'-UTR remain to be determined.

It has been reported that an ARE in the 3'-UTR regulates the expression of many types of genes, including some cytokines, immediate early genes and trophic factors [18-20]. Moreover, many families of RNA-binding proteins that specifically recognize an ARE in several genes have been identified. AUF1 consists of four splicing variants and is reported to destabilize mRNA through an ARE [22,23]. In contrast, the ELAV family members, including HuR, HuD, HuB and HuC, are suggested to enhance mRNA stabilization [24-27]. We transfected our reporter constructs containing the mouse GDNF 3'-UTR together with the AUF1 splicing variants or the ELAV family members; however, none of the ARE-binding proteins restored luciferase activity in the C6 cells transfected (unpublished data). Some stimuli (*e.g*., NGF [26], GM-CSF [27], PMA [28], LPS [29] and heat shock [30]) have been reported to stabilize mRNA through the activation of intracellular signaling pathways [26-32] and/or the modification of RNA-binding proteins [26]. We attempted to examine the effects of PMA and LPS, which were previously reported to up-regulate endogenous GDNF mRNA, but neither stimulus inhibited the suppressive effect of an ARE in the mouse GDNF 3'-UTR. As some factors, including poly(A)-binding proteins [33] and microRNAs [34], have also been reported to post-transcripionally regulate the amount of mRNA with a 3'-UTR in quantity, we investigated whether PABPc1, a poly(A)-binding protein, affects the suppressive effect of the mouse GDNF 3'-UTR. Our results show that PABPc1 overexpression does not affect this suppressive feature. Therefore, it is still unclear which factors (proteins and/or RNA molecules) participate in GDNF expression via its 3'-UTR. Using miRBase http://www.mirbase.org/ to search for microRNAs that might recognize the ARE and non-ARE regions, we found that some microRNAs (*e.g*., mmu-miR-1955-5p and mmu-miR-883a-3p) are predictied to associate with the suppressive regions within the Δ13 and Δ 5 regions, respectively. Thus, further studies on the identification and characterization of negative regulators that destabilize the GDNF mRNA in combination with AREs and other suppressive regions of the GDNF 3'-UTR are required to determine the mehanisms for regulating GDNF expression under pathophysilological conditions.

We previously characterized three distinct mouse GDNF promoters upstream of exons 1, 2 and 3 [35,36]. Brodbeck *et al. *reported that Six2, a homeobox gene, recognizes its consensus sequence in the mouse GDNF promoter 1 and potentiates its promoter activity [37]. With the exception of Six2 as a regulator of renal development [38], none of the transcriptional factors related to neuronal inflammation have been identified, although many inflammatory stimuli are reported to enhance intrinsic GDNF mRNA expression *in vivo *and *in vitro *[12-16].

## Conclusion

Our present study is the first to suggest the possible role of several regions in the 3'-UTR of the mouse GDNF gene in regulating its gene expression. Among these regions, we characterized the suppressive feature of a well-conserved A- and T-rich region (approximately 200 bp in length) in the mouse GDNF 3'-UTR. Based on the well-conserved nucleotide sequences surrounding the AREs among 11 species, the ARE of the human GDNF 3'-UTR is predicted to have a similarly suppressive role. Further characterization of the interaction of this ARE with other suppressive regions in the GDNF 3'-UTR, together with that of the GDNF promoter, will help to clarify the complex regulatory mechanisms of GDNF gene expression.

## Methods

### Construction of plasmids

For the preparation of the reporter constructs containing the 3'-UTR of mouse GDNF, various lengths of the 3'-UTR were amplified by PCR and cloned into the pGL3-Promoter vector (pGL3pro) (Promega) at the *Xba *I site that is immediately downstream of the luciferase gene. In this study, the nucleotide immediately after the stop codon in exon 4 of the mouse GDNF gene is defined as +1 (Figure 1A). The reporter constructs used in this study are shown in Figure 2. The mouse GDNF 3'-UTR was also cloned into the pGL3-Basic vector containing the mouse GDNF promoter 1 (GDNF pro) [35]. The coding region of mouse GDNF fused with the 3'-UTR was amplified by PCR and then cloned into the pcDNA3.1 vector [39].

### Cell culture and treatment

C6 cells were maintained in Ham's F-10 medium (Invitrogen) supplemented with 3% fetal bovine serum and 7% horse serum. Transfection of each construct used in this study was performed using the Lipofectamine-Plus reagent (Invitrogen) according to the manufacturer's instructions [39].

### Reporter gene assay

The reporter constructs and the pRL-TK vector, an internal control, were transfected into C6 cells in a 48-well plate. Thirty-six hours after transfection, the cells were lysed, and the luciferase activity in each lysate was measured using a Dual-Luciferase assay system (Promega). The reporter activity in each lysate was normalized to the co-transfected Renilla luciferase activity, and the results are shown as the relative luciferase activity.

### Western blot analysis

The expression levels of GDNF and EGFP in the cell lysates were estimated by Western blotting, as described previously [39]. Briefly, the transfected cells were lysed with SDS-Laemmli sample buffer [62.5 mM Tris-HCl (pH 6.8), 2% SDS and 10% glycerol], and the protein concentration of each cell lysate was determined using the Protein DC assay kit (Bio-Rad). Equal amounts of each sample were separated by 12.5% SDS-polyacrylamide electrophoresis gels, transferred onto polyvinylidene difluoride membranes (GE Healthcare Bioscience) and identified using a primary antibody against GDNF (Santa Cruz Biotechnology) or EGFP (Roche Biochemicals) and enhanced chemiluminescence (GE Healthcare Biosciences).

### Analysis of mRNA stability

The cells were harvested thirty-six hours after co-transfection with each reporter construct and an enhanced green fluorescence protein (EGFP) expression vector (pEGFP-N1) (Clontech) as an internal control. Act-D (5 μg/ml) was added to the cells 1 or 3 h before harvesting the cells, which were lysed with Trizol to extract the total RNA. The total RNA was treated with DNase (NIPPON GENE) for 15 min according to the manufacturer's instructions to degrade the contaminating reporter constructs and estimate the amount of each mRNA. After re-extraction of the treated RNA, the total RNA (0.5 μg) was converted to cDNA by reverse transcription using random ninemers to prime SuperScript III reverse transcriptase (RT) (Invitrogen), as previously described [15]. To estimate the expression level of each mRNA by RT-PCR, the specific cDNAs were mixed and amplified using PCR (Taq PCR kit, Takara). The RT-PCR primers used in this study were as follows: the luciferase sense primer, 5'-GGTGGCTCCCGCTGAATT-3'; the luciferase antisense primer, 5'-GATTTTTCTTGCGTCGAG-3'; the EGFP sense primer, 5'-ACCTACGGCAAGCTGACCCTGAA-3'; the EGFP antisense primer, 5'-CTCCAGCTTGTGCCCCAGGAT-3'; the β-actin sense primer, 5'-TGTATGCCTCTGGTCGTACC-3'; and the β-actin antisense primer, 5'-CCACGTCACACTTCATGATGG-3'. By measuring the remaining RNA to estimate the mRNA stability, we first determined the appropriate number of cycles of amplification for each gene. For the detection of luciferase, EGFP and β-actin mRNAs, the number of cycles of amplification was 28, 25 and 21, respectively. After the amplification of each gene, the products were separated by electrophoresis on 2.0% agarose gels and visualized using ethidium bromide. The fluorescence intensity of each band was scanned and quantified using NIH-Image software [15,16]. To evaluate the lower amount of luciferase mRNA derived from the pGL3-promoter vector containing the GDNF 3'UTR region, additional cycles of amplification were performed to produce fluorescence intensities in the Δ8-transfected untreated cells (0 h) that were almost similar to those derived from the pGL3pro-transfected cells. The experiments were repeated to confirm the reproducibility.

### Statistical analysis

The results are expressed as the mean ± SD of more than three cultures. The statistical analysis was performed using one way-ANOVA followed by Fischer's PLSD test. A probability of *p *< 0.01 was considered to be statistically significant.

## List of abbreviations used

GDNF: glial cell line-derived neurotrophic factor; 3'-UTR: 3'-untranslated region; ARE: AU-rich element; Act-D: Actinomycine D; PCR: polymerase chain reaction; EGFP: enhanced green fluorescence protein.

## Authors' contributions

KO conceived the study, performed the molecular genetics studies, participated in the sequence alignment and drafted the manuscripts. YH and KK participated in the study design and coordination. All of the authors read and approved the final manuscript.
